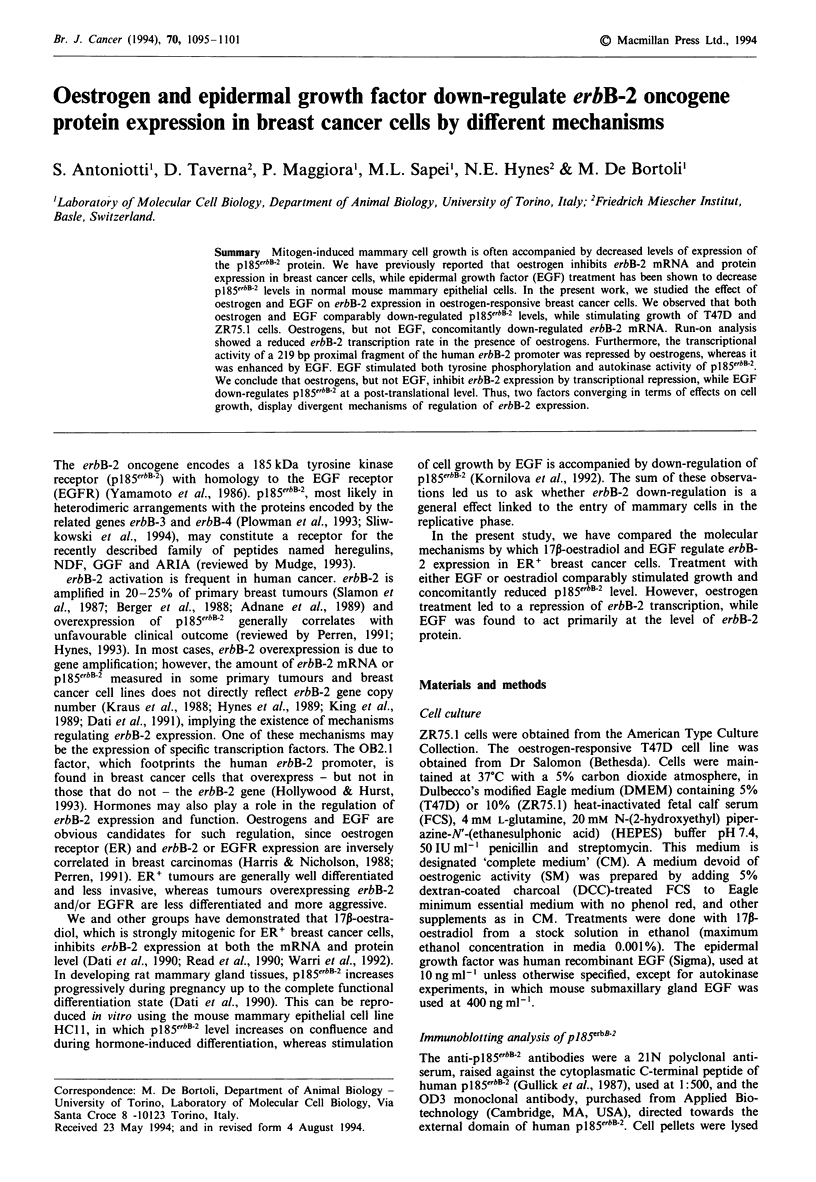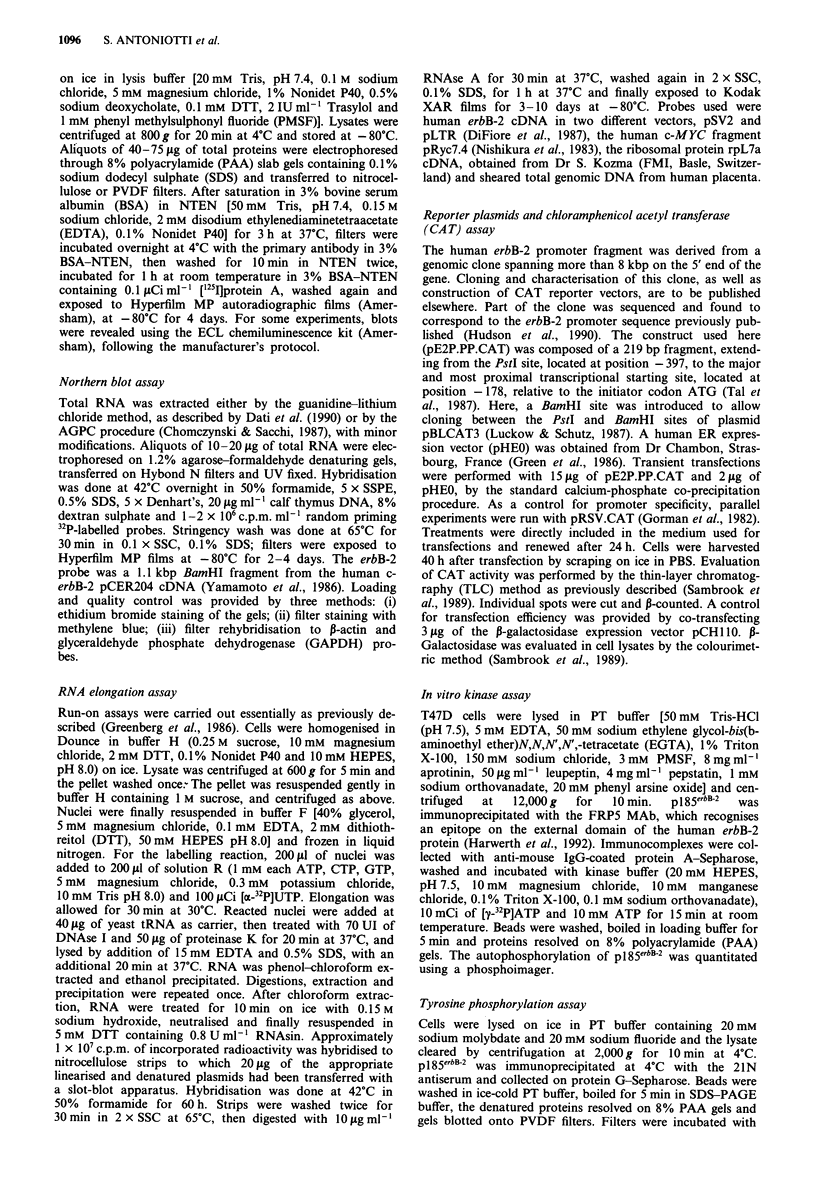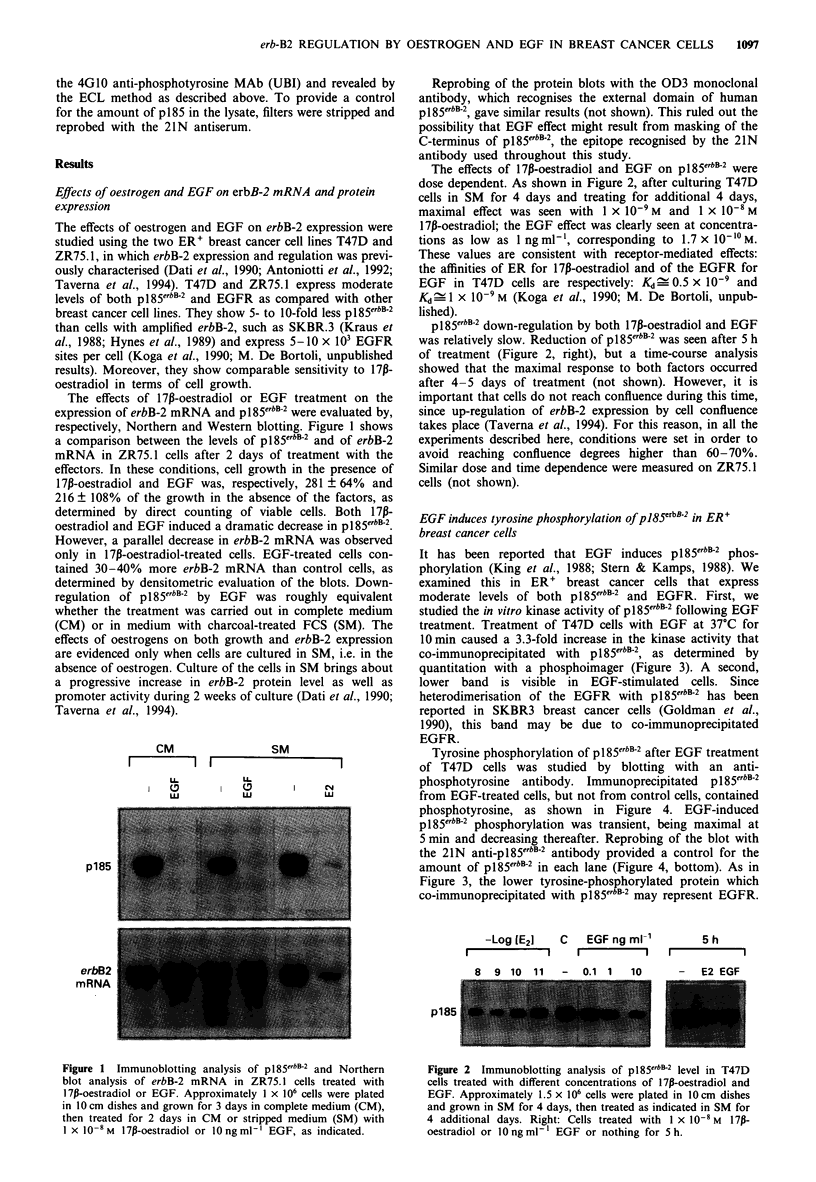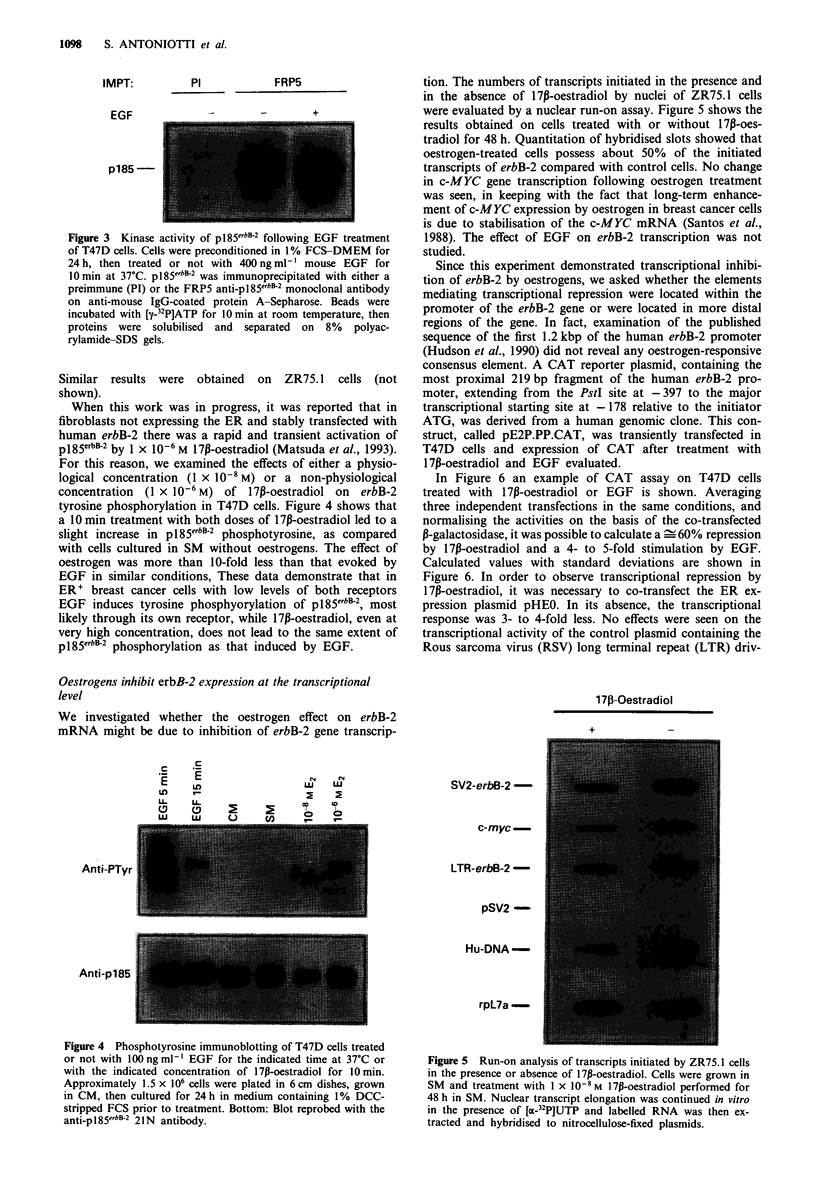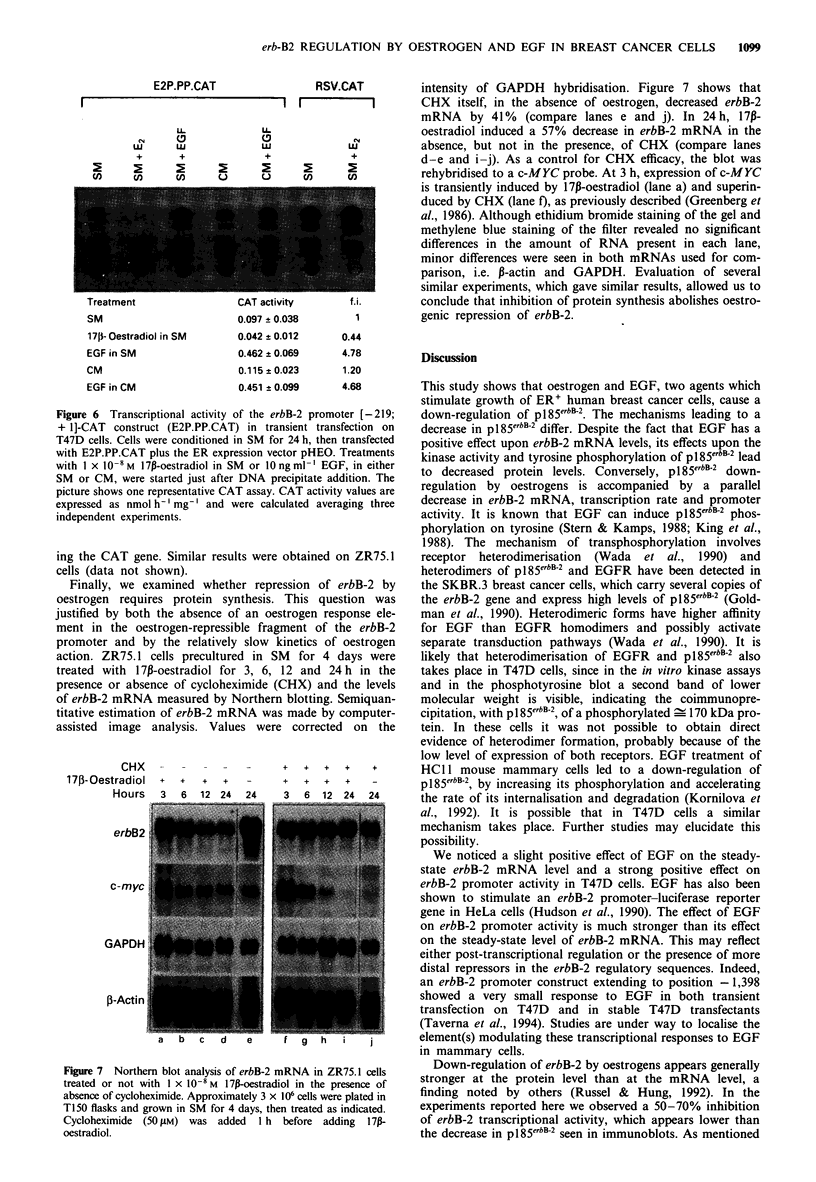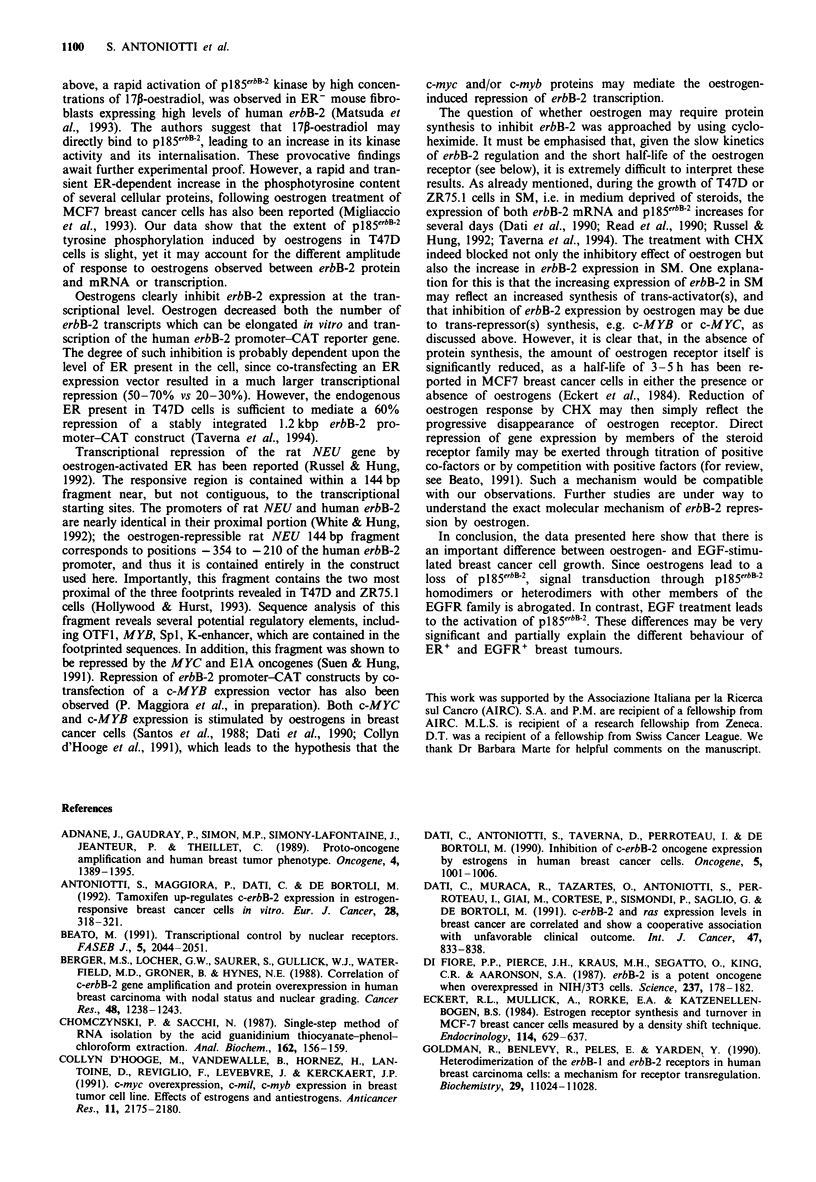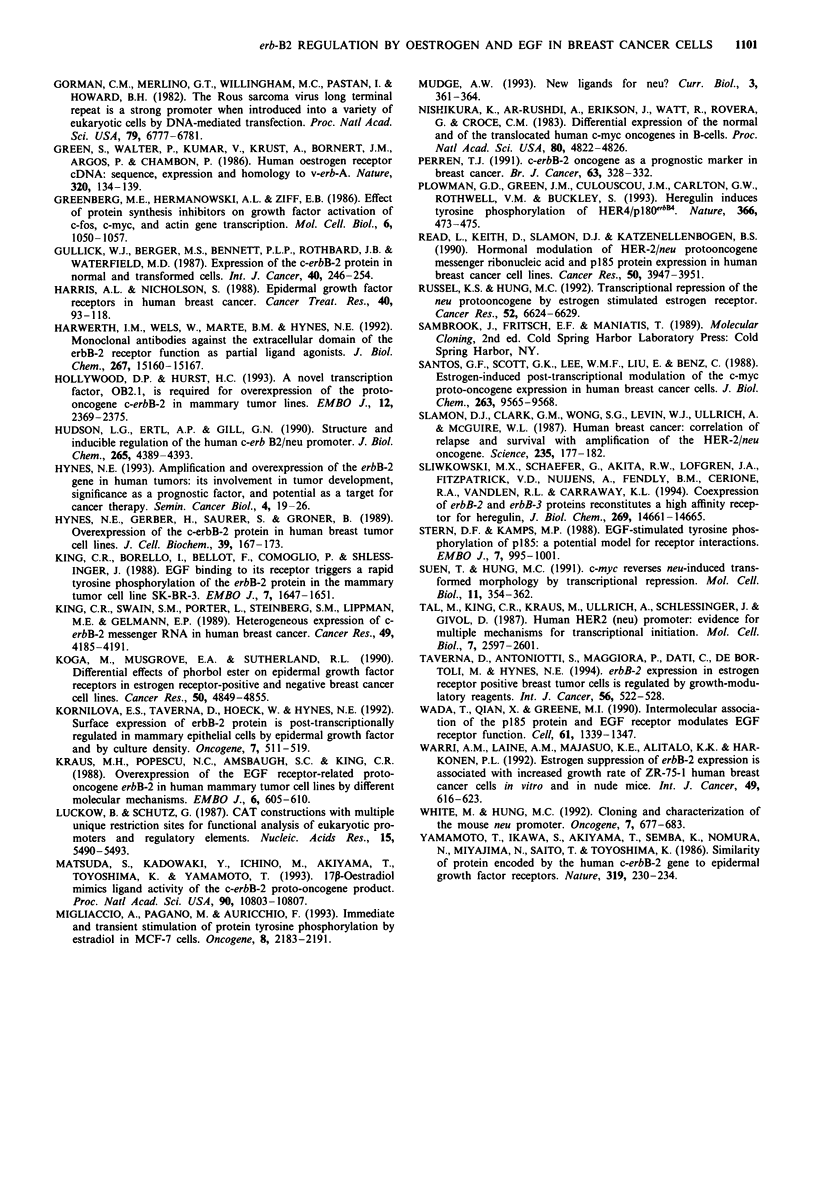# Oestrogen and epidermal growth factor down-regulate erbB-2 oncogene protein expression in breast cancer cells by different mechanisms.

**DOI:** 10.1038/bjc.1994.454

**Published:** 1994-12

**Authors:** S. Antoniotti, D. Taverna, P. Maggiora, M. L. Sapei, N. E. Hynes, M. De Bortoli

**Affiliations:** Department of Animal Biology, University of Torino, Italy.

## Abstract

**Images:**


Br. J. Cancer (1994), 70, 1095-1101 © Macmillan Press Ltd., 1994

### Oestrogen and epidermal growth factor down-regulate *erb*B-2 oncogene protein expression in breast cancer cells by different mechanisms

S. Antoniotti[1], D. Taverna[2], P. Maggiora[1], M.L. Sapei[1], N.E. Hynes[2] & M. De Bortoli[1]

[1]*Laboratory of Molecular Cell Biology, Department of Animal Biology, University of Torino, Italy;* [2]*Friedrich Miescher Institut, Basle, Switzerland.*

**Summary** Mitogen-induced mammary cell growth is often accompanied by decreased levels of expression of the $p185^{erbB\text{-}2}$ protein. We have previously reported that oestrogen inhibits *erb*B-2 mRNA and protein expression in breast cancer cells, while epidermal growth factor (EGF) treatment has been shown to decrease $p185^{erbB\text{-}2}$ levels in normal mouse mammary epithelial cells. In the present work, we studied the effect of oestrogen and EGF on *erb*B-2 expression in oestrogen-responsive breast cancer cells. We observed that both oestrogen and EGF comparably down-regulated $p185^{erbB\text{-}2}$ levels, while stimulating growth of T47D and ZR75.1 cells. Oestrogens, but not EGF, concomitantly down-regulated *erb*B-2 mRNA. Run-on analysis showed a reduced *erb*B-2 transcription rate in the presence of oestrogens. Furthermore, the transcriptional activity of a 219 bp proximal fragment of the human *erb*B-2 promoter was repressed by oestrogens, whereas it was enhanced by EGF. EGF stimulated both tyrosine phosphorylation and autokinase activity of $p185^{erbB\text{-}2}$. We conclude that oestrogens, but not EGF, inhibit *erb*B-2 expression by transcriptional repression, while EGF down-regulates $p185^{erbB\text{-}2}$ at a post-translational level. Thus, two factors converging in terms of effects on cell growth, display divergent mechanisms of regulation of *erb*B-2 expression.

The *erb*B-2 oncogene encodes a 185 kDa tyrosine kinase receptor ($p185^{erbB\text{-}2}$) with homology to the EGF receptor (EGFR) (Yamamoto *et al.*, 1986). $p185^{erbB\text{-}2}$, most likely in heterodimeric arrangements with the proteins encoded by the related genes *erb*B-3 and *erb*B-4 (Plowman *et al.*, 1993; Sliwkowski *et al.*, 1994), may constitute a receptor for the recently described family of peptides named heregulins, NDF, GGF and ARIA (reviewed by Mudge, 1993).

*erb*B-2 activation is frequent in human cancer. *erb*B-2 is amplified in 20-25% of primary breast tumours (Slamon *et al.*, 1987; Berger *et al.*, 1988; Adnane *et al.*, 1989) and overexpression of $p185^{erbB\text{-}2}$ generally correlates with unfavourable clinical outcome (reviewed by Perren, 1991; Hynes, 1993). In most cases, *erb*B-2 overexpression is due to gene amplification; however, the amount of *erb*B-2 mRNA or $p185^{erbB\text{-}2}$ measured in some primary tumours and breast cancer cell lines does not directly reflect *erb*B-2 gene copy number (Kraus *et al.*, 1988; Hynes *et al.*, 1989; King *et al.*, 1989; Dati *et al.*, 1991), implying the existence of mechanisms regulating *erb*B-2 expression. One of these mechanisms may be the expression of specific transcription factors. The OB2.1 factor, which footprints the human *erb*B-2 promoter, is found in breast cancer cells that overexpress - but not in those that do not - the *erb*B-2 gene (Hollywood & Hurst, 1993). Hormones may also play a role in the regulation of *erb*B-2 expression and function. Oestrogens and EGF are obvious candidates for such regulation, since oestrogen receptor (ER) and *erb*B-2 or EGFR expression are inversely correlated in breast carcinomas (Harris & Nicholson, 1988; Perren, 1991). $ER^+$ tumours are generally well differentiated and less invasive, whereas tumours overexpressing *erb*B-2 and/or EGFR are less differentiated and more aggressive.

We and other groups have demonstrated that 17β-oestradiol, which is strongly mitogenic for $ER^+$ breast cancer cells, inhibits *erb*B-2 expression at both the mRNA and protein level (Dati *et al.*, 1990; Read *et al.*, 1990; Warri *et al.*, 1992). In developing rat mammary gland tissues, $p185^{erbB\text{-}2}$ increases progressively during pregnancy up to the complete functional differentiation state (Dati *et al.*, 1990). This can be reproduced *in vitro* using the mouse mammary epithelial cell line HC11, in which $p185^{erbB\text{-}2}$ level increases on confluence and during hormone-induced differentiation, whereas stimulation of cell growth by EGF is accompanied by down-regulation of $p185^{erbB\text{-}2}$ (Kornilova *et al.*, 1992). The sum of these observations led us to ask whether *erb*B-2 down-regulation is a general effect linked to the entry of mammary cells in the replicative phase.

In the present study, we have compared the molecular mechanisms by which 17β-oestradiol and EGF regulate *erb*B-2 expression in $ER^+$ breast cancer cells. Treatment with either EGF or oestradiol comparably stimulated growth and concomitantly reduced $p185^{erbB\text{-}2}$ level. However, oestrogen treatment led to a repression of *erb*B-2 transcription, while EGF was found to act primarily at the level of *erb*B-2 protein.

### Materials and methods

### *Cell culture*

ZR75.1 cells were obtained from the American Type Culture Collection. The oestrogen-responsive T47D cell line was obtained from Dr Salomon (Bethesda). Cells were maintained at 37°C with a 5% carbon dioxide atmosphere, in Dulbecco's modified Eagle medium (DMEM) containing 5% (T47D) or 10% (ZR75.1) heat-inactivated fetal calf serum (FCS), 4 mM L-glutamine, 20 mM N-(2-hydroxyethyl) piperazine-N'-(ethanesulphonic acid) (HEPES) buffer pH 7.4, 50 IU $ml^{-1}$ penicillin and streptomycin. This medium is designated 'complete medium' (CM). A medium devoid of oestrogenic activity (SM) was prepared by adding 5% dextran-coated charcoal (DCC)-treated FCS to Eagle minimum essential medium with no phenol red, and other supplements as in CM. Treatments were done with 17β-oestradiol from a stock solution in ethanol (maximum ethanol concentration in media 0.001%). The epidermal growth factor was human recombinant EGF (Sigma), used at 10 ng $ml^{-1}$ unless otherwise specified, except for autokinase experiments, in which mouse submaxillary gland EGF was used at 400 ng $ml^{-1}$.

### *Immunoblotting analysis of* $p185^{erbB\text{-}2}$

The anti-$p185^{erbB\text{-}2}$ antibodies were a 21N polyclonal antiserum, raised against the cytoplasmatic C-terminal peptide of human $p185^{erbB\text{-}2}$ (Gullick *et al.*, 1987), used at 1:500, and the OD3 monoclonal antibody, purchased from Applied Biotechnology (Cambridge, MA, USA), directed towards the external domain of human $p185^{erbB\text{-}2}$. Cell pellets were lysed

Correspondence: M. De Bortoli, Department of Animal Biology - University of Torino, Laboratory of Molecular Cell Biology, Via Santa Croce 8 -10123 Torino, Italy.
Received 23 May 1994; and in revised form 4 August 1994.

on ice in lysis buffer [20 mM Tris, pH 7.4, 0.1 M sodium chloride, 5 mM magnesium chloride, 1% Nonidet P40, 0.5% sodium deoxycholate, 0.1 mM DTT, 2 IU $ml^{-1}$ Trasylol and 1 mM phenyl methylsulphonyl fluoride (PMSF)]. Lysates were centrifuged at 800 g for 20 min at 4°C and stored at −80°C. Aliquots of 40–75 μg of total proteins were electrophoresed through 8% polyacrylamide (PAA) slab gels containing 0.1% sodium dodecyl sulphate (SDS) and transferred to nitrocellulose or PVDF filters. After saturation in 3% bovine serum albumin (BSA) in NTEN [50 mM Tris, pH 7.4, 0.15 M sodium chloride, 2 mM disodium ethylenediaminetetraacetate (EDTA), 0.1% Nonidet P40] for 3 h at 37°C, filters were incubated overnight at 4°C with the primary antibody in 3% BSA–NTEN, then washed for 10 min in NTEN twice, incubated for 1 h at room temperature in 3% BSA–NTEN containing 0.1 μCi $ml^{-1}$ [$^{125}$I]protein A, washed again and exposed to Hyperfilm MP autoradiographic films (Amersham), at −80°C for 4 days. For some experiments, blots were revealed using the ECL chemiluminescence kit (Amersham), following the manufacturer's protocol.

*Northern blot assay*

Total RNA was extracted either by the guanidine–lithium chloride method, as described by Dati *et al.* (1990) or by the AGPC procedure (Chomczynski & Sacchi, 1987), with minor modifications. Aliquots of 10–20 μg of total RNA were electrophoresed on 1.2% agarose–formaldehyde denaturing gels, transferred on Hybond N filters and UV fixed. Hybridisation was done at 42°C overnight in 50% formamide, 5 × SSPE, 0.5% SDS, 5 × Denhart's, 20 μg $ml^{-1}$ calf thymus DNA, 8% dextran sulphate and 1–2 × $10^6$ c.p.m. $ml^{-1}$ random priming $^{32}$P-labelled probes. Stringency wash was done at 65°C for 30 min in 0.1 × SSC, 0.1% SDS; filters were exposed to Hyperfilm MP films at −80°C for 2–4 days. The *erb*B-2 probe was a 1.1 kbp *Bam*HI fragment from the human c-*erb*B-2 pCER204 cDNA (Yamamoto *et al.*, 1986). Loading and quality control was provided by three methods: (i) ethidium bromide staining of the gels; (ii) filter staining with methylene blue; (iii) filter rehybridisation to β-actin and glyceraldehyde phosphate dehydrogenase (GAPDH) probes.

*RNA elongation assay*

Run-on assays were carried out essentially as previously described (Greenberg *et al.*, 1986). Cells were homogenised in Dounce in buffer H (0.25 M sucrose, 10 mM magnesium chloride, 2 mM DTT, 0.1% Nonidet P40 and 10 mM HEPES, pH 8.0) on ice. Lysate was centrifuged at 600 g for 5 min and the pellet washed once. The pellet was resuspended gently in buffer H containing 1 M sucrose, and centrifuged as above. Nuclei were finally resuspended in buffer F [40% glycerol, 5 mM magnesium chloride, 0.1 mM EDTA, 2 mM dithiothreitol (DTT), 50 mM HEPES pH 8.0] and frozen in liquid nitrogen. For the labelling reaction, 200 μl of nuclei was added to 200 μl of solution R (1 mM each ATP, CTP, GTP, 5 mM magnesium chloride, 0.3 mM potassium chloride, 10 mM Tris pH 8.0) and 100 μCi [α-$^{32}$P]UTP. Elongation was allowed for 30 min at 30°C. Reacted nuclei were added at 40 μg of yeast tRNA as carrier, then treated with 70 UI of DNAse I and 50 μg of proteinase K for 20 min at 37°C, and lysed by addition of 15 mM EDTA and 0.5% SDS, with an additional 20 min at 37°C. RNA was phenol–chloroform extracted and ethanol precipitated. Digestions, extraction and precipitation were repeated once. After chloroform extraction, RNA were treated for 10 min on ice with 0.15 M sodium hydroxide, neutralised and finally resuspended in 5 mM DTT containing 0.8 U $ml^{-1}$ RNAsin. Approximately 1 × $10^7$ c.p.m. of incorporated radioactivity was hybridised to nitrocellulose strips to which 20 μg of the appropriate linearised and denatured plasmids had been transferred with a slot-blot apparatus. Hybridisation was done at 42°C in 50% formamide for 60 h. Strips were washed twice for 30 min in 2 × SSC at 65°C, then digested with 10 μg $ml^{-1}$ RNAse A for 30 min at 37°C, washed again in 2 × SSC, 0.1% SDS, for 1 h at 37°C and finally exposed to Kodak XAR films for 3–10 days at −80°C. Probes used were human *erb*B-2 cDNA in two different vectors, pSV2 and pLTR (DiFiore *et al.*, 1987), the human c-*MYC* fragment pRyc7.4 (Nishikura *et al.*, 1983), the ribosomal protein rpL7a cDNA, obtained from Dr S. Kozma (FMI, Basle, Switzerland) and sheared total genomic DNA from human placenta.

*Reporter plasmids and chloramphenicol acetyl transferase (CAT) assay*

The human *erb*B-2 promoter fragment was derived from a genomic clone spanning more than 8 kbp on the 5′ end of the gene. Cloning and characterisation of this clone, as well as construction of CAT reporter vectors, are to be published elsewhere. Part of the clone was sequenced and found to correspond to the *erb*B-2 promoter sequence previously published (Hudson *et al.*, 1990). The construct used here (pE2P.PP.CAT) was composed of a 219 bp fragment, extending from the *Pst*I site, located at position −397, to the major and most proximal transcriptional starting site, located at position −178, relative to the initiator codon ATG (Tal *et al.*, 1987). Here, a *Bam*HI site was introduced to allow cloning between the *Pst*I and *Bam*HI sites of plasmid pBLCAT3 (Luckow & Schutz, 1987). A human ER expression vector (pHE0) was obtained from Dr Chambon, Strasbourg, France (Green *et al.*, 1986). Transient transfections were performed with 15 μg of pE2P.PP.CAT and 2 μg of pHE0, by the standard calcium-phosphate co-precipitation procedure. As a control for promoter specificity, parallel experiments were run with pRSV.CAT (Gorman *et al.*, 1982). Treatments were directly included in the medium used for transfections and renewed after 24 h. Cells were harvested 40 h after transfection by scraping on ice in PBS. Evaluation of CAT activity was performed by the thin-layer chromatography (TLC) method as previously described (Sambrook *et al.*, 1989). Individual spots were cut and β-counted. A control for transfection efficiency was provided by co-transfecting 3 μg of the β-galactosidase expression vector pCH110. β-Galactosidase was evaluated in cell lysates by the colourimetric method (Sambrook *et al.*, 1989).

*In vitro kinase assay*

T47D cells were lysed in PT buffer [50 mM Tris-HCl (pH 7.5), 5 mM EDTA, 50 mM sodium ethylene glycol-*bis*(b-aminoethyl ether)*N,N,N′,N′*,-tetracetate (EGTA), 1% Triton X-100, 150 mM sodium chloride, 3 mM PMSF, 8 mg $ml^{-1}$ aprotinin, 50 μg $ml^{-1}$ leupeptin, 4 mg $ml^{-1}$ pepstatin, 1 mM sodium orthovanadate, 20 mM phenyl arsine oxide] and centrifuged at 12,000 g for 10 min. $p185^{erbB\text{-}2}$ was immunoprecipitated with the FRP5 MAb, which recognises an epitope on the external domain of the human *erb*B-2 protein (Harwerth *et al.*, 1992). Immunocomplexes were collected with anti-mouse IgG-coated protein A-Sepharose, washed and incubated with kinase buffer (20 mM HEPES, pH 7.5, 10 mM magnesium chloride, 10 mM manganese chloride, 0.1% Triton X-100, 0.1 mM sodium orthovanadate), 10 mCi of [γ-$^{32}$P]ATP and 10 mM ATP for 15 min at room temperature. Beads were washed, boiled in loading buffer for 5 min and proteins resolved on 8% polyacrylamide (PAA) gels. The autophosphorylation of $p185^{erbB\text{-}2}$ was quantitated using a phosphoimager.

*Tyrosine phosphorylation assay*

Cells were lysed on ice in PT buffer containing 20 mM sodium molybdate and 20 mM sodium fluoride and the lysate cleared by centrifugation at 2,000 g for 10 min at 4°C. $p185^{erbB\text{-}2}$ was immunoprecipitated at 4°C with the 21N antiserum and collected on protein G–Sepharose. Beads were washed in ice-cold PT buffer, boiled for 5 min in SDS–PAGE buffer, the denatured proteins resolved on 8% PAA gels and gels blotted onto PVDF filters. Filters were incubated with the 4G10 anti-phosphotyrosine MAb (UBI) and revealed by the ECL method as described above. To provide a control for the amount of p185 in the lysate, filters were stripped and reprobed with the 21N antiserum.

### Results

### *Effects of oestrogen and EGF on* erbB-2 *mRNA and protein expression*

The effects of oestrogen and EGF on *erb*B-2 expression were studied using the two $ER^+$ breast cancer cell lines T47D and ZR75.1, in which *erb*B-2 expression and regulation was previously characterised (Dati *et al.*, 1990; Antoniotti *et al.*, 1992; Taverna *et al.*, 1994). T47D and ZR75.1 express moderate levels of both $p185^{erbB\text{-}2}$ and EGFR as compared with other breast cancer cell lines. They show 5- to 10-fold less $p185^{erbB\text{-}2}$ than cells with amplified *erb*B-2, such as SKBR.3 (Kraus *et al.*, 1988; Hynes *et al.*, 1989) and express $5-10 \times 10^3$ EGFR sites per cell (Koga *et al.*, 1990; M. De Bortoli, unpublished results). Moreover, they show comparable sensitivity to 17β-oestradiol in terms of cell growth.

The effects of 17β-oestradiol or EGF treatment on the expression of *erb*B-2 mRNA and $p185^{erbB\text{-}2}$ were evaluated by, respectively, Northern and Western blotting. Figure 1 shows a comparison between the levels of $p185^{erbB\text{-}2}$ and of *erb*B-2 mRNA in ZR75.1 cells after 2 days of treatment with the effectors. In these conditions, cell growth in the presence of 17β-oestradiol and EGF was, respectively, $281 \pm 64\%$ and $216 \pm 108\%$ of the growth in the absence of the factors, as determined by direct counting of viable cells. Both 17β-oestradiol and EGF induced a dramatic decrease in $p185^{erbB\text{-}2}$. However, a parallel decrease in *erb*B-2 mRNA was observed only in 17β-oestradiol-treated cells. EGF-treated cells contained 30–40% more *erb*B-2 mRNA than control cells, as determined by densitometric evaluation of the blots. Down-regulation of $p185^{erbB\text{-}2}$ by EGF was roughly equivalent whether the treatment was carried out in complete medium (CM) or in medium with charcoal-treated FCS (SM). The effects of oestrogens on both growth and *erb*B-2 expression are evidenced only when cells are cultured in SM, i.e. in the absence of oestrogen. Culture of the cells in SM brings about a progressive increase in *erb*B-2 protein level as well as promoter activity during 2 weeks of culture (Dati *et al.*, 1990; Taverna *et al.*, 1994).

**Figure 1** Immunoblotting analysis of $p185^{erbB\text{-}2}$ and Northern blot analysis of *erb*B-2 mRNA in ZR75.1 cells treated with 17β-oestradiol or EGF. Approximately $1 \times 10^6$ cells were plated in 10 cm dishes and grown for 3 days in complete medium (CM), then treated for 2 days in CM or stripped medium (SM) with $1 \times 10^{-8}$ M 17β-oestradiol or $10\ ng\ ml^{-1}$ EGF, as indicated.

Reprobing of the protein blots with the OD3 monoclonal antibody, which recognises the external domain of human $p185^{erbB\text{-}2}$, gave similar results (not shown). This ruled out the possibility that EGF effect might result from masking of the C-terminus of $p185^{erbB\text{-}2}$, the epitope recognised by the 21N antibody used throughout this study.

The effects of 17β-oestradiol and EGF on $p185^{erbB\text{-}2}$ were dose dependent. As shown in Figure 2, after culturing T47D cells in SM for 4 days and treating for additional 4 days, maximal effect was seen with $1 \times 10^{-9}$ M and $1 \times 10^{-8}$ M 17β-oestradiol; the EGF effect was clearly seen at concentrations as low as $1\ ng\ ml^{-1}$, corresponding to $1.7 \times 10^{-10}$ M. These values are consistent with receptor-mediated effects: the affinities of ER for 17β-oestradiol and of the EGFR for EGF in T47D cells are respectively: $K_d \cong 0.5 \times 10^{-9}$ and $K_d \cong 1 \times 10^{-9}$ M (Koga *et al.*, 1990; M. De Bortoli, unpublished).

$p185^{erbB\text{-}2}$ down-regulation by both 17β-oestradiol and EGF was relatively slow. Reduction of $p185^{erbB\text{-}2}$ was seen after 5 h of treatment (Figure 2, right), but a time-course analysis showed that the maximal response to both factors occurred after 4–5 days of treatment (not shown). However, it is important that cells do not reach confluence during this time, since up-regulation of *erb*B-2 expression by cell confluence takes place (Taverna *et al.*, 1994). For this reason, in all the experiments described here, conditions were set in order to avoid reaching confluence degrees higher than 60–70%. Similar dose and time dependence were measured on ZR75.1 cells (not shown).

### *EGF induces tyrosine phosphorylation of* $p185^{erbB\text{-}2}$ *in* $ER^+$ *breast cancer cells*

It has been reported that EGF induces $p185^{erbB\text{-}2}$ phosphorylation (King *et al.*, 1988; Stern & Kamps, 1988). We examined this in $ER^+$ breast cancer cells that express moderate levels of both $p185^{erbB\text{-}2}$ and EGFR. First, we studied the *in vitro* kinase activity of $p185^{erbB\text{-}2}$ following EGF treatment. Treatment of T47D cells with EGF at 37°C for 10 min caused a 3.3-fold increase in the kinase activity that co-immunoprecipitated with $p185^{erbB\text{-}2}$, as determined by quantitation with a phosphoimager (Figure 3). A second, lower band is visible in EGF-stimulated cells. Since heterodimerisation of the EGFR with $p185^{erbB\text{-}2}$ has been reported in SKBR3 breast cancer cells (Goldman *et al.*, 1990), this band may be due to co-immunoprecipitated EGFR.

Tyrosine phosphorylation of $p185^{erbB\text{-}2}$ after EGF treatment of T47D cells was studied by blotting with an anti-phosphotyrosine antibody. Immunoprecipitated $p185^{erbB\text{-}2}$ from EGF-treated cells, but not from control cells, contained phosphotyrosine, as shown in Figure 4. EGF-induced $p185^{erbB\text{-}2}$ phosphorylation was transient, being maximal at 5 min and decreasing thereafter. Reprobing of the blot with the 21N anti-$p185^{erbB\text{-}2}$ antibody provided a control for the amount of $p185^{erbB\text{-}2}$ in each lane (Figure 4, bottom). As in Figure 3, the lower tyrosine-phosphorylated protein which co-immunoprecipitated with $p185^{erbB\text{-}2}$ may represent EGFR.

**Figure 2** Immunoblotting analysis of $p185^{erbB\text{-}2}$ level in T47D cells treated with different concentrations of 17β-oestradiol and EGF. Approximately $1.5 \times 10^6$ cells were plated in 10 cm dishes and grown in SM for 4 days, then treated as indicated in SM for 4 additional days. Right: Cells treated with $1 \times 10^{-8}$ M 17β-oestradiol or $10\ ng\ ml^{-1}$ EGF or nothing for 5 h.

**Figure 3** Kinase activity of p185$^{erbB\text{-}2}$ following EGF treatment of T47D cells. Cells were preconditioned in 1% FCS–DMEM for 24 h, then treated or not with 400 ng ml$^{-1}$ mouse EGF for 10 min at 37°C. p185$^{erbB\text{-}2}$ was immunoprecipitated with either a preimmune (PI) or the FRP5 anti-p185$^{erbB\text{-}2}$ monoclonal antibody on anti-mouse IgG-coated protein A–Sepharose. Beads were incubated with [$\gamma$-$^{32}$P]ATP for 10 min at room temperature, then proteins were solubilised and separated on 8% polyacrylamide–SDS gels.

Similar results were obtained on ZR75.1 cells (not shown).

When this work was in progress, it was reported that in fibroblasts not expressing the ER and stably transfected with human *erb*B-2 there was a rapid and transient activation of p185$^{erbB\text{-}2}$ by $1 \times 10^{-6}$ M 17*β*-oestradiol (Matsuda *et al.*, 1993). For this reason, we examined the effects of either a physiological concentration ($1 \times 10^{-8}$ M) or a non-physiological concentration ($1 \times 10^{-6}$ M) of 17*β*-oestradiol on *erb*B-2 tyrosine phosphorylation in T47D cells. Figure 4 shows that a 10 min treatment with both doses of 17*β*-oestradiol led to a slight increase in p185$^{erbB\text{-}2}$ phosphotyrosine, as compared with cells cultured in SM without oestrogens. The effect of oestrogen was more than 10-fold less than that evoked by EGF in similar conditions, These data demonstrate that in ER$^+$ breast cancer cells with low levels of both receptors EGF induces tyrosine phosphyorylation of p185$^{erbB\text{-}2}$, most likely through its own receptor, while 17*β*-oestradiol, even at very high concentration, does not lead to the same extent of p185$^{erbB\text{-}2}$ phosphorylation as that induced by EGF.

### *Oestrogens inhibit* erb*B-2 expression at the transcriptional level*

We investigated whether the oestrogen effect on *erb*B-2 mRNA might be due to inhibition of *erb*B-2 gene transcription. The numbers of transcripts initiated in the presence and in the absence of 17*β*-oestradiol by nuclei of ZR75.1 cells were evaluated by a nuclear run-on assay. Figure 5 shows the results obtained on cells treated with or without 17*β*-oestradiol for 48 h. Quantitation of hybridised slots showed that oestrogen-treated cells possess about 50% of the initiated transcripts of *erb*B-2 compared with control cells. No change in c-*MYC* gene transcription following oestrogen treatment was seen, in keeping with the fact that long-term enhancement of c-*MYC* expression by oestrogen in breast cancer cells is due to stabilisation of the c-*MYC* mRNA (Santos *et al.*, 1988). The effect of EGF on *erb*B-2 transcription was not studied.

Since this experiment demonstrated transcriptional inhibition of *erb*B-2 by oestrogens, we asked whether the elements mediating transcriptional repression were located within the promoter of the *erb*B-2 gene or were located in more distal regions of the gene. In fact, examination of the published sequence of the first 1.2 kbp of the human *erb*B-2 promoter (Hudson *et al.*, 1990) did not reveal any oestrogen-responsive consensus element. A CAT reporter plasmid, containing the most proximal 219 bp fragment of the human *erb*B-2 promoter, extending from the *Pst*I site at −397 to the major transcriptional starting site at −178 relative to the initiator ATG, was derived from a human genomic clone. This construct, called pE2P.PP.CAT, was transiently transfected in T47D cells and expression of CAT after treatment with 17*β*-oestradiol and EGF evaluated.

In Figure 6 an example of CAT assay on T47D cells treated with 17*β*-oestradiol or EGF is shown. Averaging three independent transfections in the same conditions, and normalising the activities on the basis of the co-transfected *β*-galactosidase, it was possible to calculate a ≅60% repression by 17*β*-oestradiol and a 4- to 5-fold stimulation by EGF. Calculated values with standard deviations are shown in Figure 6. In order to observe transcriptional repression by 17*β*-oestradiol, it was necessary to co-transfect the ER expression plasmid pHE0. In its absence, the transcriptional response was 3- to 4-fold less. No effects were seen on the transcriptional activity of the control plasmid containing the Rous sarcoma virus (RSV) long terminal repeat (LTR) driv-

**Figure 4** Phosphotyrosine immunoblotting of T47D cells treated or not with 100 ng ml$^{-1}$ EGF for the indicated time at 37°C or with the indicated concentration of 17*β*-oestradiol for 10 min. Approximately $1.5 \times 10^6$ cells were plated in 6 cm dishes, grown in CM, then cultured for 24 h in medium containing 1% DCC-stripped FCS prior to treatment. Bottom: Blot reprobed with the anti-p185$^{erbB\text{-}2}$ 21N antibody.

**Figure 5** Run-on analysis of transcripts initiated by ZR75.1 cells in the presence or absence of 17*β*-oestradiol. Cells were grown in SM and treatment with $1 \times 10^{-8}$ M 17*β*-oestradiol performed for 48 h in SM. Nuclear transcript elongation was continued *in vitro* in the presence of [$\alpha$-$^{32}$P]UTP and labelled RNA was then extracted and hybridised to nitrocellulose-fixed plasmids.

| Treatment | CAT activity | f.i. |
|---|---|---|
| SM | 0.097 ± 0.038 | 1 |
| 17β-Oestradiol in SM | 0.042 ± 0.012 | 0.44 |
| EGF in SM | 0.462 ± 0.069 | 4.78 |
| CM | 0.115 ± 0.023 | 1.20 |
| EGF in CM | 0.451 ± 0.099 | 4.68 |

**Figure 6** Transcriptional activity of the *erb*B-2 promoter [−219; +1]-CAT construct (E2P.PP.CAT) in transient transfection on T47D cells. Cells were conditioned in SM for 24 h, then transfected with E2P.PP.CAT plus the ER expression vector pHEO. Treatments with $1 \times 10^{-8}$ M 17β-oestradiol in SM or 10 ng ml$^{-1}$ EGF, in either SM or CM, were started just after DNA precipitate addition. The picture shows one representative CAT assay. CAT activity values are expressed as nmol h$^{-1}$ mg$^{-1}$ and were calculated averaging three independent experiments.

ing the CAT gene. Similar results were obtained on ZR75.1 cells (data not shown).

Finally, we examined whether repression of *erb*B-2 by oestrogen requires protein synthesis. This question was justified by both the absence of an oestrogen response element in the oestrogen-repressible fragment of the *erb*B-2 promoter and by the relatively slow kinetics of oestrogen action. ZR75.1 cells precultured in SM for 4 days were treated with 17β-oestradiol for 3, 6, 12 and 24 h in the presence or absence of cycloheximide (CHX) and the levels of *erb*B-2 mRNA measured by Northern blotting. Semiquantitative estimation of *erb*B-2 mRNA was made by computer-assisted image analysis. Values were corrected on the

**Figure 7** Northern blot analysis of *erb*B-2 mRNA in ZR75.1 cells treated or not with $1 \times 10^{-8}$ M 17β-oestradiol in the presence of absence of cycloheximide. Approximately $3 \times 10^6$ cells were plated in T150 flasks and grown in SM for 4 days, then treated as indicated. Cycloheximide (50 μM) was added 1 h before adding 17β-oestradiol.

intensity of GAPDH hybridisation. Figure 7 shows that CHX itself, in the absence of oestrogen, decreased *erb*B-2 mRNA by 41% (compare lanes e and j). In 24 h, 17β-oestradiol induced a 57% decrease in *erb*B-2 mRNA in the absence, but not in the presence, of CHX (compare lanes d–e and i–j). As a control for CHX efficacy, the blot was rehybridised to a c-*MYC* probe. At 3 h, expression of c-*MYC* is transiently induced by 17β-oestradiol (lane a) and superinduced by CHX (lane f), as previously described (Greenberg *et al.*, 1986). Although ethidium bromide staining of the gel and methylene blue staining of the filter revealed no significant differences in the amount of RNA present in each lane, minor differences were seen in both mRNAs used for comparison, i.e. β-actin and GAPDH. Evaluation of several similar experiments, which gave similar results, allowed us to conclude that inhibition of protein synthesis abolishes oestrogenic repression of *erb*B-2.

### Discussion

This study shows that oestrogen and EGF, two agents which stimulate growth of $ER^+$ human breast cancer cells, cause a down-regulation of $p185^{erbB\text{-}2}$. The mechanisms leading to a decrease in $p185^{erbB\text{-}2}$ differ. Despite the fact that EGF has a positive effect upon *erb*B-2 mRNA levels, its effects upon the kinase activity and tyrosine phosphorylation of $p185^{erbB\text{-}2}$ lead to decreased protein levels. Conversely, $p185^{erbB\text{-}2}$ down-regulation by oestrogens is accompanied by a parallel decrease in *erb*B-2 mRNA, transcription rate and promoter activity. It is known that EGF can induce $p185^{erbB\text{-}2}$ phosphorylation on tyrosine (Stern & Kamps, 1988; King *et al.*, 1988). The mechanism of transphosphorylation involves receptor heterodimerisation (Wada *et al.*, 1990) and heterodimers of $p185^{erbB\text{-}2}$ and EGFR have been detected in the SKBR.3 breast cancer cells, which carry several copies of the *erb*B-2 gene and express high levels of $p185^{erbB\text{-}2}$ (Goldman *et al.*, 1990). Heterodimeric forms have higher affinity for EGF than EGFR homodimers and possibly activate separate transduction pathways (Wada *et al.*, 1990). It is likely that heterodimerisation of EGFR and $p185^{erbB\text{-}2}$ also takes place in T47D cells, since in the *in vitro* kinase assays and in the phosphotyrosine blot a second band of lower molecular weight is visible, indicating the coimmunoprecipitation, with $p185^{erbB\text{-}2}$, of a phosphorylated ≅170 kDa protein. In these cells it was not possible to obtain direct evidence of heterodimer formation, probably because of the low level of expression of both receptors. EGF treatment of HC11 mouse mammary cells led to a down-regulation of $p185^{erbB\text{-}2}$, by increasing its phosphorylation and accelerating the rate of its internalisation and degradation (Kornilova *et al.*, 1992). It is possible that in T47D cells a similar mechanism takes place. Further studies may elucidate this possibility.

We noticed a slight positive effect of EGF on the steady-state *erb*B-2 mRNA level and a strong positive effect on *erb*B-2 promoter activity in T47D cells. EGF has also been shown to stimulate an *erb*B-2 promoter–luciferase reporter gene in HeLa cells (Hudson *et al.*, 1990). The effect of EGF on *erb*B-2 promoter activity is much stronger than its effect on the steady-state level of *erb*B-2 mRNA. This may reflect either post-transcriptional regulation or the presence of more distal repressors in the *erb*B-2 regulatory sequences. Indeed, an *erb*B-2 promoter construct extending to position −1,398 showed a very small response to EGF in both transient transfection on T47D and in stable T47D transfectants (Taverna *et al.*, 1994). Studies are under way to localise the element(s) modulating these transcriptional responses to EGF in mammary cells.

Down-regulation of *erb*B-2 by oestrogens appears generally stronger at the protein level than at the mRNA level, a finding noted by others (Russel & Hung, 1992). In the experiments reported here we observed a 50–70% inhibition of *erb*B-2 transcriptional activity, which appears lower than the decrease in $p185^{erbB\text{-}2}$ seen in immunoblots. As mentioned above, a rapid activation of p185$^{erbB-2}$ kinase by high concentrations of 17β-oestradiol, was observed in ER$^-$ mouse fibroblasts expressing high levels of human *erb*B-2 (Matsuda *et al.*, 1993). The authors suggest that 17β-oestradiol may directly bind to p185$^{erbB-2}$, leading to an increase in its kinase activity and its internalisation. These provocative findings await further experimental proof. However, a rapid and transient ER-dependent increase in the phosphotyrosine content of several cellular proteins, following oestrogen treatment of MCF7 breast cancer cells has also been reported (Migliaccio *et al.*, 1993). Our data show that the extent of p185$^{erbB-2}$ tyrosine phosphorylation induced by oestrogens in T47D cells is slight, yet it may account for the different amplitude of response to oestrogens observed between *erb*B-2 protein and mRNA or transcription.

Oestrogens clearly inhibit *erb*B-2 expression at the transcriptional level. Oestrogen decreased both the number of *erb*B-2 transcripts which can be elongated *in vitro* and transcription of the human *erb*B-2 promoter–CAT reporter gene. The degree of such inhibition is probably dependent upon the level of ER present in the cell, since co-transfecting an ER expression vector resulted in a much larger transcriptional repression (50–70% *vs* 20–30%). However, the endogenous ER present in T47D cells is sufficient to mediate a 60% repression of a stably integrated 1.2 kbp *erb*B-2 promoter–CAT construct (Taverna *et al.*, 1994).

Transcriptional repression of the rat *NEU* gene by oestrogen-activated ER has been reported (Russel & Hung, 1992). The responsive region is contained within a 144 bp fragment near, but not contiguous, to the transcriptional starting sites. The promoters of rat *NEU* and human *erb*B-2 are nearly identical in their proximal portion (White & Hung, 1992); the oestrogen-repressible rat *NEU* 144 bp fragment corresponds to positions −354 to −210 of the human *erb*B-2 promoter, and thus it is contained entirely in the construct used here. Importantly, this fragment contains the two most proximal of the three footprints revealed in T47D and ZR75.1 cells (Hollywood & Hurst, 1993). Sequence analysis of this fragment reveals several potential regulatory elements, including OTF1, *MYB*, Sp1, K-enhancer, which are contained in the footprinted sequences. In addition, this fragment was shown to be repressed by the *MYC* and E1A oncogenes (Suen & Hung, 1991). Repression of *erb*B-2 promoter–CAT constructs by co-transfection of a c-*MYB* expression vector has also been observed (P. Maggiora *et al.*, in preparation). Both c-*MYC* and c-*MYB* expression is stimulated by oestrogens in breast cancer cells (Santos *et al.*, 1988; Dati *et al.*, 1990; Collyn d'Hooge *et al.*, 1991), which leads to the hypothesis that the c-*myc* and/or c-*myb* proteins may mediate the oestrogen-induced repression of *erb*B-2 transcription.

The question of whether oestrogen may require protein synthesis to inhibit *erb*B-2 was approached by using cycloheximide. It must be emphasised that, given the slow kinetics of *erb*B-2 regulation and the short half-life of the oestrogen receptor (see below), it is extremely difficult to interpret these results. As already mentioned, during the growth of T47D or ZR75.1 cells in SM, i.e. in medium deprived of steroids, the expression of both *erb*B-2 mRNA and p185$^{erbB-2}$ increases for several days (Dati *et al.*, 1990; Read *et al.*, 1990; Russel & Hung, 1992; Taverna *et al.*, 1994). The treatment with CHX indeed blocked not only the inhibitory effect of oestrogen but also the increase in *erb*B-2 expression in SM. One explanation for this is that the increasing expression of *erb*B-2 in SM may reflect an increased synthesis of trans-activator(s), and that inhibition of *erb*B-2 expression by oestrogen may be due to trans-repressor(s) synthesis, e.g. c-*MYB* or c-*MYC*, as discussed above. However, it is clear that, in the absence of protein synthesis, the amount of oestrogen receptor itself is significantly reduced, as a half-life of 3–5 h has been reported in MCF7 breast cancer cells in either the presence or absence of oestrogens (Eckert *et al.*, 1984). Reduction of oestrogen response by CHX may then simply reflect the progressive disappearance of oestrogen receptor. Direct repression of gene expression by members of the steroid receptor family may be exerted through titration of positive co-factors or by competition with positive factors (for review, see Beato, 1991). Such a mechanism would be compatible with our observations. Further studies are under way to understand the exact molecular mechanism of *erb*B-2 repression by oestrogen.

In conclusion, the data presented here show that there is an important difference between oestrogen- and EGF-stimulated breast cancer cell growth. Since oestrogens lead to a loss of p185$^{erbB-2}$, signal transduction through p185$^{erbB-2}$ homodimers or heterodimers with other members of the EGFR family is abrogated. In contrast, EGF treatment leads to the activation of p185$^{erbB-2}$. These differences may be very significant and partially explain the different behaviour of ER$^+$ and EGFR$^+$ breast tumours.

This work was supported by the Associazione Italiana per la Ricerca sul Cancro (AIRC). S.A. and P.M. are recipient of a fellowship from AIRC. M.L.S. is recipient of a research fellowship from Zeneca. D.T. was a recipient of a fellowship from Swiss Cancer League. We thank Dr Barbara Marte for helpful comments on the manuscript.

### References

ADNANE, J., GAUDRAY, P., SIMON, M.P., SIMONY-LAFONTAINE, J., JEANTEUR, P. & THEILLET, C. (1989). Proto-oncogene amplification and human breast tumor phenotype. *Oncogene*, **4**, 1389–1395.

ANTONIOTTI, S., MAGGIORA, P., DATI, C. & DE BORTOLI, M. (1992). Tamoxifen up-regulates c-*erb*B-2 expression in estrogen-responsive breast cancer cells *in vitro*. *Eur. J. Cancer*, **28**, 318–321.

BEATO, M. (1991). Transcriptional control by nuclear receptors. *FASEB J.*, **5**, 2044–2051.

BERGER, M.S., LOCHER, G.W., SAURER, S., GULLICK, W.J., WATERFIELD, M.D., GRONER, B. & HYNES, N.E. (1988). Correlation of c-*erb*B-2 gene amplification and protein overexpression in human breast carcinoma with nodal status and nuclear grading. *Cancer Res.*, **48**, 1238–1243.

CHOMCZYNSKI, P. & SACCHI, N. (1987). Single-step method of RNA isolation by the acid guanidinium thiocyanate–phenol–chloroform extraction. *Anal. Biochem.*, **162**, 156–159.

COLLYN D'HOOGE, M., VANDEWALLE, B., HORNEZ, H., LANTOINE, D., REVIGLIO, F., LEVEBVRE, J. & KERCKAERT, J.P. (1991). c-*myc* overexpression, c-*mil*, c-*myb* expression in breast tumor cell line. Effects of estrogens and antiestrogens. *Anticancer Res.*, **11**, 2175–2180.

DATI, C., ANTONIOTTI, S., TAVERNA, D., PERROTEAU, I. & DE BORTOLI, M. (1990). Inhibition of c-*erb*B-2 oncogene expression by estrogens in human breast cancer cells. *Oncogene*, **5**, 1001–1006.

DATI, C., MURACA, R., TAZARTES, O., ANTONIOTTI, S., PERROTEAU, I., GIAI, M., CORTESE, P., SISMONDI, P., SAGLIO, G. & DE BORTOLI, M. (1991). c-*erb*B-2 and *ras* expression levels in breast cancer are correlated and show a cooperative association with unfavorable clinical outcome. *Int. J. Cancer*, **47**, 833–838.

DI FIORE, P.P., PIERCE, J.H., KRAUS, M.H., SEGATTO, O., KING, C.R. & AARONSON, S.A. (1987). *erb*B-2 is a potent oncogene when overexpressed in NIH/3T3 cells. *Science*, **237**, 178–182.

ECKERT, R.L., MULLICK, A., RORKE, E.A. & KATZENELLENBOGEN, B.S. (1984). Estrogen receptor synthesis and turnover in MCF-7 breast cancer cells measured by a density shift technique. *Endocrinology*, **114**, 629–637.

GOLDMAN, R., BENLEVY, R., PELES, E. & YARDEN, Y. (1990). Heterodimerization of the *erb*B-1 and *erb*B-2 receptors in human breast carcinoma cells: a mechanism for receptor transregulation. *Biochemistry*, **29**, 11024–11028.

GORMAN, C.M., MERLINO, G.T., WILLINGHAM, M.C., PASTAN, I. & HOWARD, B.H. (1982). The Rous sarcoma virus long terminal repeat is a strong promoter when introduced into a variety of eukaryotic cells by DNA-mediated transfection. *Proc. Natl Acad. Sci. USA*, **79**, 6777-6781.

GREEN, S., WALTER, P., KUMAR, V., KRUST, A., BORNERT, J.M., ARGOS, P. & CHAMBON, P. (1986). Human oestrogen receptor cDNA: sequence, expression and homology to v-*erb*-A. *Nature*, **320**, 134-139.

GREENBERG, M.E., HERMANOWSKI, A.L. & ZIFF, E.B. (1986). Effect of protein synthesis inhibitors on growth factor activation of c-fos, c-myc, and actin gene transcription. *Mol. Cell. Biol.*, **6**, 1050-1057.

GULLICK, W.J., BERGER, M.S., BENNETT, P.L.P., ROTHBARD, J.B. & WATERFIELD, M.D. (1987). Expression of the c-*erb*B-2 protein in normal and transformed cells. *Int. J. Cancer*, **40**, 246-254.

HARRIS, A.L. & NICHOLSON, S. (1988). Epidermal growth factor receptors in human breast cancer. *Cancer Treat. Res.*, **40**, 93-118.

HARWERTH, I.M., WELS, W., MARTE, B.M. & HYNES, N.E. (1992). Monoclonal antibodies against the extracellular domain of the erbB-2 receptor function as partial ligand agonists. *J. Biol. Chem.*, **267**, 15160-15167.

HOLLYWOOD, D.P. & HURST, H.C. (1993). A novel transcription factor, OB2.1, is required for overexpression of the proto-oncogene c-*erb*B-2 in mammary tumor lines. *EMBO J.*, **12**, 2369-2375.

HUDSON, L.G., ERTL, A.P. & GILL, G.N. (1990). Structure and inducible regulation of the human c-*erb* B2/neu promoter. *J. Biol. Chem.*, **265**, 4389-4393.

HYNES, N.E. (1993). Amplification and overexpression of the *erb*B-2 gene in human tumors: its involvement in tumor development, significance as a prognostic factor, and potential as a target for cancer therapy. *Semin. Cancer Biol.*, **4**, 19-26.

HYNES, N.E., GERBER, H., SAURER, S. & GRONER, B. (1989). Overexpression of the c-erbB-2 protein in human breast tumor cell lines. *J. Cell. Biochem.*, **39**, 167-173.

KING, C.R., BORELLO, I., BELLOT, F., COMOGLIO, P. & SHLESSINGER, J. (1988). EGF binding to its receptor triggers a rapid tyrosine phosphorylation of the *erb*B-2 protein in the mammary tumor cell line SK-BR-3. *EMBO J.*, **7**, 1647-1651.

KING, C.R., SWAIN, S.M., PORTER, L., STEINBERG, S.M., LIPPMAN, M.E. & GELMANN, E.P. (1989). Heterogeneous expression of c-*erb*B-2 messenger RNA in human breast cancer. *Cancer Res.*, **49**, 4185-4191.

KOGA, M., MUSGROVE, E.A. & SUTHERLAND, R.L. (1990). Differential effects of phorbol ester on epidermal growth factor receptors in estrogen receptor-positive and negative breast cancer cell lines. *Cancer Res.*, **50**, 4849-4855.

KORNILOVA, E.S., TAVERNA, D., HOECK, W. & HYNES, N.E. (1992). Surface expression of erbB-2 protein is post-transcriptionally regulated in mammary epithelial cells by epidermal growth factor and by culture density. *Oncogene*, **7**, 511-519.

KRAUS, M.H., POPESCU, N.C., AMSBAUGH, S.C. & KING, C.R. (1988). Overexpression of the EGF receptor-related proto-oncogene *erb*B-2 in human mammary tumor cell lines by different molecular mechanisms. *EMBO J.*, **6**, 605-610.

LUCKOW, B. & SCHUTZ, G. (1987). CAT constructions with multiple unique restriction sites for functional analysis of eukaryotic promoters and regulatory elements. *Nucleic. Acids Res.*, **15**, 5490-5493.

MATSUDA, S., KADOWAKI, Y., ICHINO, M., AKIYAMA, T., TOYOSHIMA, K. & YAMAMOTO, T. (1993). 17β-Oestradiol mimics ligand activity of the c-*erb*B-2 proto-oncogene product. *Proc. Natl Acad. Sci. USA*, **90**, 10803-10807.

MIGLIACCIO, A., PAGANO, M. & AURICCHIO, F. (1993). Immediate and transient stimulation of protein tyrosine phosphorylation by estradiol in MCF-7 cells. *Oncogene*, **8**, 2183-2191.

MUDGE, A.W. (1993). New ligands for neu? *Curr. Biol.*, **3**, 361-364.

NISHIKURA, K., AR-RUSHDI, A., ERIKSON, J., WATT, R., ROVERA, G. & CROCE, C.M. (1983). Differential expression of the normal and of the translocated human c-myc oncogenes in B-cells. *Proc. Natl Acad. Sci. USA*, **80**, 4822-4826.

PERREN, T.J. (1991). c-*erb*B-2 oncogene as a prognostic marker in breast cancer. *Br. J. Cancer*, **63**, 328-332.

PLOWMAN, G.D., GREEN, J.M., CULOUSCOU, J.M., CARLTON, G.W., ROTHWELL, V.M. & BUCKLEY, S. (1993). Heregulin induces tyrosine phosphorylation of HER4/p180$^{erbB4}$. *Nature*, **366**, 473-475.

READ, L., KEITH, D., SLAMON, D.J. & KATZENELLENBOGEN, B.S. (1990). Hormonal modulation of HER-2/*neu* protooncogene messenger ribonucleic acid and p185 protein expression in human breast cancer cell lines. *Cancer Res.*, **50**, 3947-3951.

RUSSEL, K.S. & HUNG, M.C. (1992). Transcriptional repression of the *neu* protooncogene by estrogen stimulated estrogen receptor. *Cancer Res.*, **52**, 6624-6629.

SAMBROOK, J., FRITSCH, E.F. & MANIATIS, T. (1989). *Molecular Cloning*, 2nd ed. Cold Spring Harbor Laboratory Press: Cold Spring Harbor, NY.

SANTOS, G.F., SCOTT, G.K., LEE, W.M.F., LIU, E. & BENZ, C. (1988). Estrogen-induced post-transcriptional modulation of the c-myc proto-oncogene expression in human breast cancer cells. *J. Biol. Chem.*, **263**, 9565-9568.

SLAMON, D.J., CLARK, G.M., WONG, S.G., LEVIN, W.J., ULLRICH, A. & MCGUIRE, W.L. (1987). Human breast cancer: correlation of relapse and survival with amplification of the HER-2/*neu* oncogene. *Science*, **235**, 177-182.

SLIWKOWSKI, M.X., SCHAEFER, G., AKITA, R.W., LOFGREN, J.A., FITZPATRICK, V.D., NUIJENS, A., FENDLY, B.M., CERIONE, R.A., VANDLEN, R.L. & CARRAWAY, K.L. (1994). Coexpression of *erbB-2* and *erbB-3* proteins reconstitutes a high affinity receptor for heregulin, *J. Biol. Chem.*, **269**, 14661-14665.

STERN, D.F. & KAMPS, M.P. (1988). EGF-stimulated tyrosine phosphorylation of p185: a potential model for receptor interactions. *EMBO J.*, **7**, 995-1001.

SUEN, T. & HUNG, M.C. (1991). c-*myc* reverses *neu*-induced transformed morphology by transcriptional repression. *Mol. Cell. Biol.*, **11**, 354-362.

TAL, M., KING, C.R., KRAUS, M., ULLRICH, A., SCHLESSINGER, J. & GIVOL, D. (1987). Human HER2 (neu) promoter: evidence for multiple mechanisms for transcriptional initiation. *Mol. Cell. Biol.*, **7**, 2597-2601.

TAVERNA, D., ANTONIOTTI, S., MAGGIORA, P., DATI, C., DE BORTOLI, M. & HYNES, N.E. (1994). *erbB-2* expression in estrogen receptor positive breast tumor cells is regulated by growth-modulatory reagents. *Int. J. Cancer*, **56**, 522-528.

WADA, T., QIAN, X. & GREENE, M.I. (1990). Intermolecular association of the p185 protein and EGF receptor modulates EGF receptor function. *Cell*, **61**, 1339-1347.

WARRI, A.M., LAINE, A.M., MAJASUO, K.E., ALITALO, K.K. & HARKONEN, P.L. (1992). Estrogen suppression of *erb*B-2 expression is associated with increased growth rate of ZR-75-1 human breast cancer cells *in vitro* and in nude mice. *Int. J. Cancer*, **49**, 616-623.

WHITE, M. & HUNG, M.C. (1992). Cloning and characterization of the mouse *neu* promoter. *Oncogene*, **7**, 677-683.

YAMAMOTO, T., IKAWA, S., AKIYAMA, T., SEMBA, K., NOMURA, N., MIYAJIMA, N., SAITO, T. & TOYOSHIMA, K. (1986). Similarity of protein encoded by the human c-*erb*B-2 gene to epidermal growth factor receptors. *Nature*, **319**, 230-234.